# Intentional Recreational Activities of Daily Living and Well‐Being in the General Population and in Psychosomatic Patients, Before and After Treatment

**DOI:** 10.1111/sjop.70043

**Published:** 2025-11-10

**Authors:** Michael Linden, Christopher Arnold, Barbara Lieberei, Matthias Rose, Beate Muschalla

**Affiliations:** ^1^ Charité University Medicine Berlin Research Group Psychosomatic Rehabilitation Berlin Germany; ^2^ Dr Ebel Rehabilitation Centre for Psychosomatic Medicine Potsdam Germany; ^3^ Technische Universität Braunschweig, Department of Psychotherapy and Diagnostics Braunschweig Germany

**Keywords:** behavioral activation, motivation, psychosomatics, psychotherapy, volition

## Abstract

Mental illness can affect activities of daily living, instrumental activities of daily living, and recreational activities of daily living (RADL, e.g., sports, hobbies). RADL can have positive effects on psychological well‐being, and can therefore also intentionally be used to improve one's well‐being (IRADL, intentional recreational activities of daily living). In a German representative and a convenience sample, 2522 participants and 213 patients were asked about their use of IRADL. The psychosomatic patients were asked pre and post a 5‐week stay in a psychosomatic hospital. More than half of the participants in the representative study, 2/3 of the pretreatment, and over 91% of the posttreatment psychosomatic sample indicated at least one recreational activity that they use to deliberately improve psychological well‐being. Most people in the general public use recreational activities in a well‐being‐promoting manner. Psychosomatic patients use this option more frequently. Psychosomatic treatment helps to increase the motivation to use recreational activities.

**Summary**

- Many people of the general public use recreational activities in order to promote their mental well‐being.
- This is especially the case for people who suffer from mental disorders.
- Psychosomatic treatment is a meaningful way to enhance the frequency of the use of recreational activities.

## 1 Introduction

Effects of illness are not only symptoms but also limitations in capacities and activities and restrictions in participation, as outlined by the International Classification of Impairment Disability, and Health, ICF (WHO 2001). Capacities to perform “activities of daily living,” like whether a person can eat, bathe, and dress without help, decide about the degree of autonomy, and the need for assistance. Such basic activities can be measured and quantified with ADL scales (activities of daily living) (Katz et al. 1963).

Next, there are “instrumental activities of daily living (IADL).” They describe whether a person is able to perform complex activities that are needed for mastery of daily living. Examples are housework, laundry, shopping, managing money, and taking medicines (Lawton and Brody 1969).

Finally, there are “recreational activities of daily living (RADL).” These activities encompass sport, relaxation, hobbies, meeting with friends, vacation, and many others, which support well‐being and life satisfaction (Linden et al. 2009; Schmiedeberg and Schröder 2017).

These RADL can be further distinguished into intentionally used recreational activities to improve well‐being (IRADL, intentional recreational activities of daily living). The usage of these particular activities is specifically aimed at counteracting subjectively negative mental states, distress, or for general distraction. Persons go jogging, for a walk, visit the sauna, go to the cinema, or participate in other cultural activities, as this is experienced as pleasant, soothing, and helps to distance themselves from negative feelings. Engaging in IRADL can enhance subjective well‐being and, as explained in the positive activity model (Lyubomirsky and Layous 2013), is influenced by the conditions of positive activities and characteristics of the person. Well‐being regarding IRADL is therefore, for example, guided by emotions, the dosage and variety of activities, as well as motivational characteristics which are essential for intentional activities (Jenkins et al. 2021; Lyubomirsky and Layous 2013).

There are many studies that show that mental disorders are often associated with a reduction in the overall level of activities. This preferably pertains to recreational activities. This then can start a vicious circle, as inactivity can further impair mental well‐being (Pascoe et al. 2020; Teychenne et al. 2020; Wolf et al. 2021). In addition to this, it is also known for long that social contacts, self‐care, hobbies, etc., are important elements of salutogenesis and healthy living in general (Fancourt et al. 2021; Li et al. 2019; Linden and Weig 2009; Sala et al. 2019; Santini et al. 2022).

Beneficial effects of RADL have also been described across many mental disorders (Bone et al. 2022; Fancourt et al. 2021; Jacob et al. 2020; Jeong and Park 2020; Lee et al. 2023; Markotić et al. 2020; Sala et al. 2019; Tough et al. 2017; Żok et al. 2022). Therefore, RADL is explicitly used as a therapeutic intervention. It has been one of the earliest therapeutic interventions in behavior therapy to increase activities in the treatment of depressive disorders by use of pleasant event lists (Lewinsohn and Libet 1972). There is also the slogan “running through the mind,” to show the beneficial effects of sport on mental well‐being (Greist et al. 1979). For inpatients and outpatients, there are manualized treatments under the heading of “behavioral activation” (Lejuez et al. 2001; Martell et al. 2021; Stein et al. 2021). In psychiatric and psychosomatic hospitals, activation also has a major role in treatment programs. Patients are encouraged to participate in occupational therapy, sports therapy, social encounters, structured daily routines, and cultural activities (DRV 2015; Möckel and Treusch 2020). Particularly, an essential component of psychosomatic treatments is the strengthening of recreational activities, such as sport, exercise in general, social interaction, and other recreational activities. Psychosomatic diseases, which are primarily seen in psychosomatic care, are characterized by a complex interplay between mental and physical aspects. As mentioned, treatment approaches are usually focused on both aspects. These approaches not only promote physical and psychological recovery but also strengthen patients' resources for long‐term therapeutic success, as psychosomatic diseases often benefit from extensive care that addresses both mental and physical domains. Patients in psychosomatic clinics are often characterized by specific features such as chronic stress, somatic symptom disorders, or emotional dysregulation. It is precisely these characteristics that make them receptive to approaches that focus on active participation and self‐efficacy, as recreational activities can help them regain a sense of well‐being and strengthen their resilience (Lejuez et al. 2001; Martell et al. 2021; Möckel and Treusch 2020; Stein et al. 2021).

In spite of this manyfold research, an open question is to what degree IRADL is used by people in the general population and by psychosomatic patients, and whether this rate changes in the course of targeted treatment.

## 2 Materials and Methods

### 2.1 Assessment of Intentional Activities

Participants filled in the IRADL scale (intentional recreational activities of daily living), a short version of the RADL‐ICF scale (Linden 2018). It covers 12 leisure time/recreational activities: “social encounters,” “television and internet,” “relaxation and silence,” “cooking and eating,” “excursions, traveling, and ventures,” “listening to and making music,” “hobbies,” “wellness and self‐care,” “reading and literature,” “sports and exercise,” “activities in nature,” “enjoying cultural activities.” Patients indicate with yes and no: “I deliberately and regularly do this so that I feel better.”

### 2.2 General Population

Face‐to‐face interviews were done in a sample that is representative of the general population in Germany. The investigation was conducted by a professional social research company (USUMA GmbH Berlin) with many years of expertise in population‐based representative surveys. The sampling process was three‐staged: First, sampling areas were selected by random sampling. USUMA works with 250 sample areas throughout Germany, whereby 10 interviews are conducted per area. Secondly, the random selection of households was done in these areas, based on on‐site inspection. In a third stage, the interviewer identified all households and selected one interview person per household, by means of a predetermined random procedure using the “Swedish key.” Exclusion criteria included a minimum age of 16 years, and only households within Germany were considered. There were no other exclusion criteria.

Interviewers were specifically trained and followed operating instructions. Informed consent of participants was obtained prior to the data collection. The interviews were done on site. Socio‐demographic data and indicators of physical or mental disorders were assessed. Participants filled in the IRADL scale.

### 2.3 Psychosomatic Inpatients

Patients were assessed in a psychosomatic rehabilitation hospital (Dr Ebel Rehabilitation Centre for Psychosomatic Medicine, Potsdam, Germany). Patients are admitted for inpatient treatment on their own initiative or upon request by health and pension insurance when their ability to work is endangered. Accordingly, patients are suffering from all kinds of mental disorders such as major depressive, anxiety, somatoform, adjustment, or other disorders. Inpatient treatment includes single and group psychotherapy, medical and pharmacotherapy, and social care. A major part of the treatment regimen is to activate patients. Sport therapy encourages patients to exercise and engage in group matches, dancing, or walking. Occupational therapy motivates patients to remember old hobbies or start new ones, and to test relaxing activities. In individual and group psychotherapy, patients are encouraged to get in contact with others, plan joint activities, and look out for self‐care, which includes activation in general. Apart from these structured therapies, the setting of the hospital encourages activities in general. There is a lake nearby, forests, playgrounds, and relaxing areas. The goal is to bring patients out of their lethargy and counteract negative emotional states.

At the beginning of the hospital stay, all patients are seen by a senior therapist and asked to fill in several questionnaires. Patients were included in our study if they scored ≥ 18 on the ADNM‐8 scale (Adjustment Disorder—New Module 8) (Maercker et al. 2007), which indicates an increased degree of burdens and distress. Patients filled in the study scales again at the end of the inpatient stay after 5 weeks on average. Every patient admitted to the hospital was eligible to voluntarily participate in the study if the ADNM‐8 scale score was sufficiently high. There were no other exclusion criteria.

### 2.4 Ethical Considerations

The procedure plan of both studies was reviewed and confirmed by an ethics committee. Participation in both the representative study and the rehabilitation study was voluntary.

For the representative study, after selecting a target person, the research background of the study, as well as the voluntary nature and the right to withdraw participation at a later date, were explained verbally, and written informed consent was obtained prior to any data collection. In addition to an accompanying official letter regarding the research project, all those interested in participating were given a written data protection declaration, which guaranteed strict confidentiality for all information provided in the questionnaire and contained all important information on the handling of the collected data in accordance with the provisions of the General Data Protection Regulation (“DSG‐VO”), as well as information on the exact handling of personal data. The data was anonymized so that it could not be traced back to individual participants.

For the rehabilitation sample, information on the study was provided prior to data collection. All potential participants were informed about the voluntary nature of participation, the handling of personal data in accordance with current standards, and the anonymization of the data. Patients were also informed that they could withdraw from the study at any time. Written informed consent was obtained in advance of any data collection. The data were anonymized so that no identifying information could be drawn about individual participants.

### 2.5 Statistics

Statistical analyses were done with SPSS, version 22.0.0.

Where applicable, analysis of variance (ANOVA) was used. If the requirements were not met (mainly normal distribution, homoscedasticity), Kruskal–Wallis H‐tests were used due to the less strict requirements. For the pre‐post comparison of the total score, a paired t‐test was used. For the calculation of pre‐post differences of specific activities, McNemar test was calculated. For the comparison of the representational and prepsychosomatic study χ^2^‐Test was used. The threshold for significance was determined < 0.05.

## 3 Results

A total of 2522 participants were examined in the representative sample, and 213 patients in the rehabilitation hospital.

In the representative sample, the mean age was 49 years (range 16–96, SD = 17.65) with 50.1% females, and in the psychosomatic sample 52.94 years (range 29–70, SD = 8.33), with 76.1% females. The rehabilitation study corresponds to the usual gender ratio and average age of other studies in the field (Keller et al. 2021).

In the representative sample, 52.8% of participants indicated that they deliberately and regularly use at least one activity to feel better, with an average number of 1.37 (SD = 1.99) activities. *In the p*re‐treatment psychosomatic sample, this rate was 67%, with an average number of 1.92 (SD = 2.27) activities. Posttreatment, the rate increased to 91.1%, with an average of 3.99 (SD = 2.85) activities. Differences between the pretreatment psychosomatic sample and the representative group (H (1) = 20.23; *p* = < 0.001) as well as between the posttreatment psychosomatic sample and the representative group (H (1) = 196.85; *p* = < 0.001) were both significant. Also, the rate increased significantly from pre to posttreatment (*t* (170) = −8.79, *p* = < 0.001).

**FIGURE 1:**
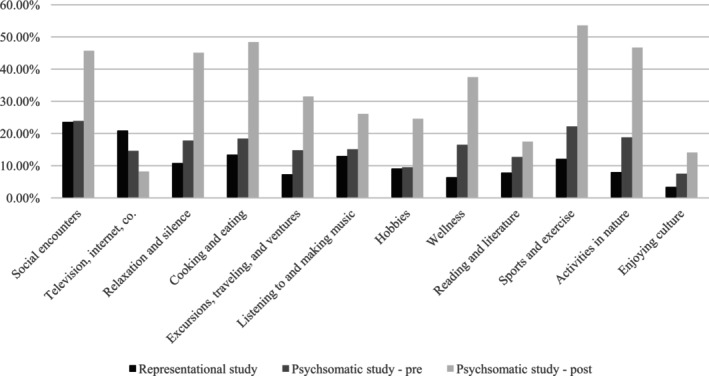
Frequency of participants selecting “I will deliberately and regularly do this so that I feel better” for each activity of the IRADL scale, divided into different study groups.

As shown in Table 1 and Figure 1, the most frequent activities in the representative sample are social contacts (23.6%) and TV (20.9%), followed by cooking/eating (13.4%), listening to music (13.0%), sport (12.0%), relaxation (10.8%), and hobbies (9.1%).

**TABLE 1:** Frequency of participants selecting “I will deliberately and regularly do this so that I feel better” for each activity of the IRADL scale, divided into different study groups.

Variable	RS	PPS	TPS	RS – PSS (**χ²‐Test, sig**.)	PPS ‐ TPS (McNemar‐Test)
Social encounters	23.60%	23.90%	45.70%	**χ^2^ (1) = 0.01 *p* = 0.921**	*p* = < 0.001
Television, internet, co.	20.90%	14.60%	8.20%	**χ^2^ (1) = 4.75 *p* = 0.029**	*p* = 0.029
Relaxation and silence	10.80%	17.80%	45.10%	**χ^2^ (1) = 9.58 *p* = 0.002**	*p* = < 0.001
Cooking and eating	13.40%	18.40%	48.40%	**χ^2^ (1) = 4.02 *p* = 0.045**	*p* = < 0.001
Excursions, traveling, and ventures	7.30%	14.80%	31.50%	**χ^2^ (1) = 15.32 *p* = < 0.001**	*p* = < 0.001
Listening to and making music	13%	15.10%	26.10%	**χ^2^ (1) = 0.773 *p* = 0.379**	*p* = 0.004
Hobbies	9.10%	9.50%	24.60%	**χ^2^ (1) = 0.037 *p* = 0.848**	*p* = < 0.001
Wellness	6.40%	16.50%	37.50%	**χ^2^ (1) = 30.102 *p* = < 0.001**	*p* = < 0.001
Reading and literature	7.80%	12.70%	17.50%	**χ^2^ (1) = 6.295 *p* = 0.012**	*p* = 0.392
Sports and exercise	12.10%	22.20%	53.60%	**χ^2^ (1) = 17.678 *p* = < 0.001**	*p* = < 0.001
Activities in nature	8%	18.80%	46.70%	**χ^2^ (1) = 27.908 *p* = < 0.001**	*p* = < 0.001
Enjoying culture	3.40%	7.50%	14.10%	**χ^2^ (1) = 8.933 *p* = 0.003**	*p* = 0.017

*Note:* RS: Representational Study; PPS: Pretreatment psychosomatic study; TPS: Posttreatment psychosomatic study.

The rank order of activities in the pretreatment patient sample also starts with social contacts (23.9%), followed by cooking/eating (18.4%), relaxation (17.8%), and wellness (16.5%).

*In the posttreatment sample, the most frequent are sport (54.0%), cooking/eating (48.4%), activities in nature (46.7%), social encounters (45.7%), and wellness (37.5%), while TV is last (8.0%)*.

Table 2 shows the differences comparing gender and persons under and over the age of 50. Female participants (*M* = 1.67, SD = 2.24) showed more activities than male participants (*M* = 1.13, SD = 1.71), (*F* (1, 2681) = 48.575; *p* = < 0.001). This is true for all types of activities except TV and hobbies.

**TABLE 2:** Frequency of participants selecting “I will deliberately and regularly do this so that I feel better” divided into gender and age groups.

Variable	Female	Male		< 50 years old	> 50 years old	
Social encounters	26.2%	20.8%	**χ** ^**2**^ **(1) = 10.667 *p* = 0.001**	29.2%	18.5%	**χ** ^**2**^ **(1) = 43.070 *p* = < 0.001**
Television, internet, co.	20.5%	20.4%	**χ** ^**2**^ **(1) = 0.005 *p* = 0.944**	21.5%	19.4%	**χ** ^**2**^ **(1) = 1.932 *p* = 0.165**
Relaxation and silence	14.9%	7.5%	**χ** ^**2**^ **(1) = 37.251 *p* = < 0.001**	10.3%	12.4%	**χ** ^**2**^ **(1) = 2.88 *p* = 0.09**
Cooking and eating	19.7%	7.4%	**χ** ^**2**^ **(1) = 87.472 *p* = < 0.001**	14.4%	13.3%	**χ** ^**2**^ **(1) = 0.737 *p* = 0.391**
Excursions, traveling, and ventures	9.2%	6.4%	**χ** ^**2**^ **(1) = 7.533 *p* = 0.006**	8%	7.7%	**χ** ^**2**^ **(1) = 0.105 *p* = 0.746**
Listening to and making music	14.6%	11.5%	**χ** ^**2**^ **(1) = 5.754 *p* = 0.016**	17.8%	8.9%	**χ** ^**2**^ **(1) = 47.577 *p* = < 0.001**
Hobbies	9.7%	8.4%	**χ** ^**2**^ **(1) = 1.285 *p* = 0.257**	10%	8.3%	**χ** ^**2**^ **(1) = 2.402 *p* = 0.121**
Wellness	10%	4.1%	**χ** ^**2**^ **(1) = 34.583 *p* = < 0.001**	6.6%	7.7%	**χ** ^**2**^ **(1) = 1.053 *p* = 0.305**
Reading and literature	11.8%	4.3%	**χ** ^**2**^ **(1) = 50.689 *p* = < 0.001**	7.4%	8.9%	**χ** ^**2**^ **(1) = 2.112 *p* = 0.146**
Sports and exercise	13%	12.7%	**χ** ^**2**^ **(1) = 0.042 *p* = 0.838**	15.6%	10.4%	**χ** ^**2**^ **(1) = 16.197 *p* = < 0.001**
Activities in nature	11.2%	6.3%	**χ** ^**2**^ **(1) = 20.563 *p* = < 0.001**	7.7%	10%	**χ** ^**2**^ **(1) = 4.337 *p* = 0.037**
Enjoying culture	5.1%	2.2%	**χ** ^**2**^ **(1) = 15.906 *p* = < 0.001**	**3.8%**	3.7%	**χ** ^**2**^ **(1) = 0.016 *p* = 0.90**

Participants under the age of 50 (*M* = 1.53, SD = 2.02) showed in general more activities than participants over the age of 50 (*M* = 1.30, SD = 2.01), (*F* (1, 2685) = 8.668; *p* = 0.003), which is due to selected activity types, such as social encounters, listening to music, sport. Older persons indicate more activities in nature.

## 4 Discussion

This is to our knowledge the first study that compares representative data from the general population with answers from psychosomatic patients before and after comprehensive inpatient treatment.

The first result is that more than half of the people in the general population indicate that they currently use at least one activity to deliberately improve their personal well‐being. This is not the rate of activities in general. People mostly watch TV without the intention of improving their emotional state. It is something different if one turns on the TV because they want to distract from some hassles and nuisances. There are data showing that RADL is rather commonly used (Schmiedeberg and Schröder 2017). Our data show that they are also regularly used as IRADL in a health‐promoting manner.

The rank order of activities in the representative sample provides insight into the nature of IRADL. Social contacts and TV are frequent in general, and it seems plausible that they are also suitable for distraction. This is equally true for the less frequent activities, such as listening to music or eating. These are mostly passive activities. Sports or other active activities are less frequent. The least frequent are cultural activities, which may be explained by the fact that these require some special expertise.

The second result is that psychosomatic patients also use IRADL to promote mental well‐being. This is different from the reduction of the overall rate of activity in patients with mental illnesses (Nagata et al. 2020; Schuch et al. 2017; Yang et al. 2018). People with mental disorders use certain methods to improve their mental status, be it for distraction, feeling excitement, or vaguely “feeling happy.”

Looking at gender, females generally engage more in IRADL. The biggest differences are found for specific activities like “cooking/eating” (19.7% vs. 7.4%), “reading” (11.8% vs. 4.3%), and “wellness” (10% vs. 4.1%). What people use and experience as a break from current problems seems to, in part, depend on their gender. Interest and the frequency of engaging in these activities apart from well‐being promotion may be generally lower in males (Hall et al. 2017; Wolfson et al. 2021).

Another distinction can be made between participants below and above the age of 50. Below the age of 50, participants engage more often in IRADL than older participants. “Listening to and making music” (17.8% vs. 8.9%), “social encounters” (29.2% vs. 18.5%), and “sports and exercise” (15.6% vs. 10.4%) were significantly more often used by people under the age of 50, while activities in nature (7.7% vs. 10.0%) are preferred by the older ones. This is plausible and consistent with the general findings emphasizing less usage of these activities in the older generations (Kasar and Karaman 2021; Rakovac and Pedisic 2022), though these activities can also be advantageous for mental well‐being in the elderly (Newman and Zainal 2020; Oladi et al. 2023; Wang et al. 2023).

Patients obviously experience that they can partially influence their current mental state by engaging in activities. Two‐thirds of the patients indicate that activities are a method of self‐help. Of interest is that, in contrast to the general reduction in RADL, the rate of IRADL is higher in patients with mental disorders. Patients obviously use activities of various kinds to influence their well‐being. Nevertheless, the overall rank order of activities is similar in both samples, with the greatest positive differences for sports, activities in nature, wellness, and a negative difference for TV. This suggests that patients actively try to counteract their blues.

An open question that arises is to what degree these activities actually enhance well‐being, and if some activities are more beneficial for the promotion of well‐being. Since characteristics of activities and the individual are essential for well‐being, the impact may vary depending on different activities (Jenkins et al. 2021; Lyubomirsky and Layous 2013). While the intention to use activities for well‐being promotion is relevant, it is unclear whether individual activities in fact achieve the desired well‐being benefit. In addition, it is important to emphasize that other factors, such as socio‐economic status, cultural background, and other factors, also influence the relationship (Giles‐Corti and Donovan 2002; Petersen et al. 2021). More literature on this topic is also needed for the intentional use of RADL.

The differences between the general population or pretreatment patients on the one hand and the posttreatment patients on the other hand are quantitatively and qualitatively marked. In the general population, 52.8% point to at least one item with “I will deliberately and regularly do this so that I feel better,” and 67% in pretreatment patients, which rises to 91.1% postlevel. Posttreatment, patients show significantly elevated rates for all activities, with the difference of TV, which is markedly reduced. Patients clearly have learned during the inpatient stay that they can and should use activities to influence their well‐being. As the rank order suggests, they have also learned that active actions are better than passive ones. Post treatment, the most frequent activities are sports, cooking/eating, activities in nature, and social encounters. However, there is also self‐nurturing in the form of relaxation and wellness. Watching TV, which is leading in the general population, is now the last item. This speaks for the effectiveness of the inpatient rehabilitation program in regard to behavioral activation, which is in accordance with other findings (Martell et al. 2021; Naji Esfahani et al. 2020).

The results of the study show that well‐being must be actively pursued, underlining the need for motivational programs during rehabilitation. This points to the fact that intentionally chosen activities, such as social, creative, or physical activity, play a central role in creating and maintaining well‐being, which also underlines broader theories on the enhancement of well‐being (Lyubomirsky et al. 2005). For this reason, well‐being requires targeted actions to positively shape one's life. Specific programs for certain groups of people need support in improving the use of recreational activities, such as psychosomatic patients (Möckel and Treusch 2020). The results emphasize the need to focus psychosomatic rehabilitation on motivation building for recreational activities.

## 5 Limitations

We used self‐ratings and asked for IRADL only. The binary distinction of the usage of IRADL limits the results, as different implementation levels of IRADL may differ and were not controlled. No statement can be made about the frequency with which recreational activities were used, which may have an influence on well‐being. Further studies should also assess RADL, be more detailed in the description of activities, ask for usage frequencies, and also look for correlates and predictors of the global rate as well as single activities. Longitudinal follow‐up studies are needed to further research the impact of IRADL in therapeutic care as well as on the general public.

## Author Contributions

**Michael Linden**: conceptualization of the study, supervision, writing, editing. **Christopher Arnold:** data analyses, data curation, writing, editing. **Barbara Lieberei:** provision of clinical data, supervision, review. **Matthias Rose:** principal investigator. **Beate Muschalla:** provision of epidemiological data, conceptualization, writing.

## Data Availability

The data that support the findings of this study are available from the corresponding author upon reasonable request.

## References

[sjop70043-bib-0001] Bone, J. K. , F. Bu , M. E. Fluharty , E. Paul , J. K. Sonke , and D. Fancourt . 2022. “Engagement in Leisure Activities and Depression in Older Adults in the United States: Longitudinal Evidence From the Health and Retirement Study.” Social Science & Medicine 294: 114703. 10.1016/j.socscimed.2022.114703. 35032746 PMC8850653

[sjop70043-bib-0002] Deutsche Rentenversicherung . 2015. Klassifikation Therapeutischer Leistungen in Der Medizinischen Rehabilitation. Ausgabe.

[sjop70043-bib-0003] Fancourt, D. , H. Aughterson , S. Finn , E. Walker , and A. Steptoe . 2021. “How Leisure Activities Affect Health: A Narrative Review and Multi‐Level Theoretical Framework of Mechanisms of Action.” Lancet Psychiatry 8, no. 4: 329–339. 10.1016/S2215-0366(20)30384-9. 33581775 PMC7613155

[sjop70043-bib-0004] Giles‐Corti, B. , and R. J. Donovan . 2002. “Socioeconomic Status Differences in Recreational Physical Activity Levels and Real and Perceived Access to a Supportive Physical Environment.” Preventive Medicine 35, no. 6: 601–611. 10.1006/pmed.2002.1115. 12460528

[sjop70043-bib-0005] Greist, J. H. , M. H. Klein , R. R. Eischens , J. Faris , A. S. Gurman , and W. P. Morgan . 1979. “Running Through Your Mind.” In The Coming Age of Psychosomatics. Pergamon. 10.1016/B978-0-08-023736-7.50010-1.

[sjop70043-bib-0006] Hall, J. L. , K. M. Kelly , L. F. Burmeister , and J. A. Merchant . 2017. “Workforce Characteristics and Attitudes Regarding Participation in Worksite Wellness Programs.” American Journal of Health Promotion 31, no. 5: 391–400. 10.4278/ajhp.140613-QUAN-2. 26730552

[sjop70043-bib-0007] Jacob, L. , M. A. Tully , Y. Barnett , et al. 2020. “The Relationship Between Physical Activity and Mental Health in a Sample of the UK Public: A Cross‐Sectional Study During the Implementation of COVID‐19 Social Distancing Measures.” Mental Health and Physical Activity 19: 100345. 10.1016/j.mhpa.2020.100345. 32834833 PMC7378001

[sjop70043-bib-0008] Jenkins, M. , S. Houge Mackenzie , K. Hodge , E. A. Hargreaves , J. R. Calverley , and C. Lee . 2021. “Physical Activity and Psychological Well‐Being During the COVID‐19 Lockdown: Relationships With Motivational Quality and Nature Contexts.” Frontiers in Sports and Active Living 3: 637576. 10.3389/fspor.2021.637576. 33733237 PMC7959839

[sjop70043-bib-0009] Jeong, E. H. , and J. H. Park . 2020. “The Relationship Among Leisure Activities, Depression and Quality of Life in Community‐Dwelling Elderly Koreans.” Gerontology and Geriatric Medicine 6: 2333721420923449. 10.1177/2333721420923449. 32551330 PMC7281882

[sjop70043-bib-0010] Kasar, K. S. , and E. Karaman . 2021. “Life in Lockdown: Social Isolation, Loneliness and Quality of Life in the Elderly During the COVID‐19 Pandemic: A Scoping Review.” Geriatric Nursing 42, no. 5: 1222–1229. 10.1016/j.gerinurse.2021.03.010. 33824008 PMC8566023

[sjop70043-bib-0011] Katz, S. , A. A. Ford , R. W. Moskowitz , B. A. Jackson , and M. W. Jaffe . 1963. “Studies of Illness in the Aged.” JAMA 185: 914–919. 10.1001/jama.1963.03060120024016. 14044222

[sjop70043-bib-0012] Keller, F. M. , A. Dahmen , C. Derksen , L. Kötting , and S. Lippke . 2021. “Psychosomatic Rehabilitation Patients and the General Population During COVID‐19: Online Cross‐Sectional and Longitudinal Study of Digital Trainings and Rehabilitation Effects.” JMIR Mental Health 8, no. 8: e30610. 10.2196/30610. 34270444 PMC8396547

[sjop70043-bib-0013] Lawton, M. P. , and E. M. Brody . 1969. “Assessment of Older People: Self‐Maintaining and Instrumental Activities of Daily Living.” Gerontologist 9, no. 1: 179–186. 5349366

[sjop70043-bib-0014] Lee, D. J. , G. B. Yu , and M. J. Sirgy . 2023. “The Dual Effects of Passion on Leisure Wellbeing: Toward a Theory of Engagement in Diverse Leisure Activities.” Applied Research in Quality of Life 19: 155–177. 10.1007/s11482-023-10235-3.

[sjop70043-bib-0015] Lejuez, C. W. , D. R. Hopko , and S. D. Hopko . 2001. “A Brief Behavioral Activation Treatment for Depression: Treatment Manual.” Behavior Modification 25, no. 2: 255–286. 10.1177/0145445501252005. 11317637

[sjop70043-bib-0017] Lewinsohn, P. M. , and J. Libet . 1972. “Pleasant Events, Activity Schedules, and Depressions.” Journal of Abnormal Psychology 79, no. 3: 291–295. 10.1037/h0033207. 5033370

[sjop70043-bib-0018] Li, Z. , J. Dai , N. Wu , Y. Jia , J. Gao , and H. Fu . 2019. “Effect of Long Working Hours on Depression and Mental Well‐Being Among Employees in Shanghai: The Role of Having Leisure Hobbies.” International Journal of Environmental Research and Public Health 16, no. 24: 4980. 10.3390/ijerph16244980. 31817855 PMC6950581

[sjop70043-bib-0019] Linden, M. 2018. “RADL‐ICF‐Skala. International Classification of Functioning, Disability and Health ‐ Recreational Activities of Daily Living‐Skala.” In Leibniz‐Institut für Psychologie (ZPID). ZPID.

[sjop70043-bib-0020] Linden, M. , G. Gehrke , and B. Geiselmann . 2009. “Profiles of Recreational Activities of Daily Living (RADL) in Patients With Mental Disorders.” Psychiatria Danubina 21: 490–496. 10.23668/psycharchives.2331. 19935482

[sjop70043-bib-0021] Linden, M. , and W. Weig , eds. 2009. Salutotherapie. Deutscher Ärzteverlag.

[sjop70043-bib-0022] Lyubomirsky, S. , and K. Layous . 2013. “How Do Simple Positive Activities Increase Well‐Being?” Current Directions in Psychological Science 22, no. 1: 57–62. 10.1177/0963721412469809.

[sjop70043-bib-0023] Lyubomirsky, S. , K. M. Sheldon , and D. Schkade . 2005. “Pursuing Happiness: The Architecture of Sustainable Change.” Review of General Psychology 9, no. 2: 111–131. 10.1037/1089-2680.9.2.11.

[sjop70043-bib-0024] Maercker, A. , F. Einsle , and V. Köllner . 2007. “Adjustment Disorders as Stress Response Syndromes: A New Diagnostic Concept and Its Exploration in a Medical Sample.” Psychopathology 40, no. 3: 135–146. 10.1159/000099290. 17284941

[sjop70043-bib-0025] Markotić, V. , V. Pokrajčić , M. Babić , et al. 2020. “The Positive Effects of Running on Mental Health.” Psychiatria Danubina 32, no. 2: 233–235. 32970641

[sjop70043-bib-0026] Martell, C. R. , S. Dimidjian , and R. Herman‐Dunn . 2021. Behavioral Activation for Depression: A Clinician's Guide. Guilford Publications.

[sjop70043-bib-0027] Möckel, L. , and Y. Treusch . 2020. “Effektivität Von Ergotherapie Bei Menschen Mit Depression‐Eine Metaanalyse.” Ergoscience 15, no. 3: 90–97. 10.2443/skv-s-2020-54020200301.

[sjop70043-bib-0028] Nagata, S. , G. Townley , E. Brusilovskiy , and M. S. Salzer . 2020. “Community Participation Differences Between Adults With or Without Serious Mental Illness.” Psychiatric Services 71, no. 11: 1191–1194. 10.1176/appi.ps.201900608. 32966173

[sjop70043-bib-0029] Naji Esfahani, F. , M. Seirafi , and A. Kraskian Mujembari . 2020. “The Effectiveness of Behavioral Activation Intervention on Increasing Self‐Care Behaviors and Life Expectancy in the Elderly.” Aging Psychology 6, no. 2: 93–105.

[sjop70043-bib-0030] Newman, M. G. , and N. H. Zainal . 2020. “The Value of Maintaining Social Connections for Mental Health in Older People.” Lancet Public Health 5, no. 1: e12–e13. 10.1016/S2468-2667(19)30253-1. 31910976 PMC7261393

[sjop70043-bib-0031] Oladi, E. , M. Esfahani , A. Azimkhani , and S. Asan . 2023. “The Effect of Physical Activity and Selected Games in Leisure Time on the Feeling of Loneliness, Self‐Efficacy, and Life Expectancy of Elderly Women.” Akdeniz Spor Bilimleri Dergisi 6, no. 2: 670–681. 10.38021/asbid.1227714.

[sjop70043-bib-0032] Pascoe, M. , A. P. Bailey , M. Craike , et al. 2020. “Physical Activity and Exercise in Youth Mental Health Promotion: A Scoping Review.” BMJ Open Sport & Exercise Medicine 6, no. 1: e000677. 10.1136/bmjsem-2019-000677. PMC7010991 32095272

[sjop70043-bib-0033] Petersen, C. B. , M. Bekker‐Jeppesen , M. Aadahl , and C. J. Lau . 2021. “Participation in Recreational Activities Varies With Socioeconomic Position and Is Associated With Self‐Rated Health and Well‐Being.” Preventive Medicine Reports 24: 101610. 10.1016/j.pmedr.2021.101610. 34976666 PMC8684004

[sjop70043-bib-0034] Rakovac, M. , and Z. Pedisic . 2022. “Physical Activity and Sport Participation in the European Union,”.

[sjop70043-bib-0035] Sala, G. , D. Jopp , F. Gobet , et al. 2019. “The Impact of Leisure Activities on Older Adults' Cognitive Function, Physical Function, and Mental Health.” PLoS One 14, no. 11: e0225006. 10.1371/journal.pone.0225006. 31703115 PMC6839878

[sjop70043-bib-0036] Santini, Z. I. , V. Koushede , C. Hinrichsen , et al. 2022. “Challenging Leisure Activities and Mental Health: Are They More Beneficial for Some People Than for Others?” Mental Health and Social Inclusion 26, no. 1: 34–46. 10.1108/MHSI-06-2021-0033.

[sjop70043-bib-0037] Schmiedeberg, C. , and J. Schröder . 2017. “Leisure Activities and Life Satisfaction: An Analysis With German Panel Data.” Applied Research in Quality of Life 12: 137–151. 10.1007/s11482-016-9458-7.

[sjop70043-bib-0038] Schuch, F. , D. Vancampfort , J. Firth , et al. 2017. “Physical Activity and Sedentary Behavior in People With Major Depressive Disorder: A Systematic Review and Meta‐Analysis.” Journal of Affective Disorders 210: 139–150. 10.1016/j.jad.2016.10.050. 28033521

[sjop70043-bib-0039] Stein, A. T. , E. Carl , P. Cuijpers , E. Karyotaki , and J. A. Smits . 2021. “Looking Beyond Depression: A Meta‐Analysis of the Effect of Behavioral Activation on Depression, Anxiety, and Activation.” Psychological Medicine 51, no. 9: 1491–1504. 10.1017/S0033291720000239. 32138802

[sjop70043-bib-0040] Teychenne, M. , R. L. White , J. Richards , F. B. Schuch , S. Rosenbaum , and J. A. Bennie . 2020. “Do We Need Physical Activity Guidelines for Mental Health: What Does the Evidence Tell Us?” Mental Health and Physical Activity 18: 100315. 10.1016/j.mhpa.2019.100315.

[sjop70043-bib-0041] Tough, H. , J. Siegrist , and C. Fekete . 2017. “Social Relationships, Mental Health and Wellbeing in Physical Disability: A Systematic Review.” BMC Public Health 17, no. 1: 414. 10.1186/s12889-017-4308-6. 28482878 PMC5422915

[sjop70043-bib-0042] Wang, S. , G. Kuan , Y. C. Kueh , K. Zhou , Y. Wang , and M. Zhao . 2023. “Research Progress on Tai Chi Intervention for Treating Depression Disorder Among the Elderly.” In Advancing Sports and Exercise via Innovation: Proceedings of the 9th Asian South Pacific Association of Sport Psychology International Congress (ASPASP). Springer Nature Singapore. 10.1007/978-981-19-8159-3_32.

[sjop70043-bib-0043] Wolf, S. , B. Seiffer , J. M. Zeibig , et al. 2021. “Is Physical Activity Associated With Less Depression and Anxiety During the COVID‐19 Pandemic? A Rapid Systematic Review.” Sports Medicine 51, no. 8: 1771–1783. 10.1007/s40279-021-01468-z. 33886101 PMC8060908

[sjop70043-bib-0044] Wolfson, J. A. , Y. Ishikawa , C. Hosokawa , K. Janisch , J. Massa , and D. M. Eisenberg . 2021. “Gender Differences in Global Estimates of Cooking Frequency Prior to COVID‐19.” Appetite 161: 105117. 10.1016/j.appet.2021.105117. 33460693 PMC8491109

[sjop70043-bib-0045] World Health Organization . 2001. International Classification of Functioning, Disability and Health (ICF). WHO.

[sjop70043-bib-0046] Yang, J. , E. C. Park , S. A. Lee , et al. 2018. “The Association Between Social Contacts and Depressive Symptoms Among Elderly Koreans.” Psychiatry Investigation 15, no. 9: 861–868. 10.30773/pi.2018.06.28.1. 30184614 PMC6166029

[sjop70043-bib-0047] Żok, A. , J. Zapała , and E. Baum . 2022. “Activities Based on Yoga Techniques in Psychiatric Treatment in Poland in a Historical Context.” Psychiatria Polska 56: 1405–1416. 10.12740/pp/onlinefirst/128776. 37098206

